# Interaction with the *N*-acetylgalactosamine moiety facilitates pore formation by Cry4Aa toxin, thereby increasing its toxicity

**DOI:** 10.1128/aem.00714-26

**Published:** 2026-09-15

**Authors:** Mohammad Tofazzal Hossain Howlader, Taiyo Tsutsui, Kouichi Saito, Tohru Hayakawa

**Affiliations:** 1 Department of Entomology, Bangladesh Agricultural University 54492, Mymensingh, Bangladesh; 2 Applied Chemistry and Biotechnology Program, School of Engineering, Okayama University 12997, Okayama, Japan; 3 Graduate School of Interdisciplinary Science and Engineering in Health Systems, Okayama University 509597, Okayama, Japan; Universidad de los Andes, Bogotá, Colombia

**Keywords:** *Bacillus thuringiensis*ser serovar *israelensis*, Cry4Aa toxin, *Culex pipiens *mosquito larvae, interaction with *N*-acetylgalactosamine

## Abstract

Cry4Aa is a mosquito-larvicidal toxin produced by *Bacillus thuringiensis* serovar *israelensis*. Cry4Aa exhibits a characteristic architecture comprising three domains (I, II, and III) and functions as a pore-forming toxin, consistent with many previously reported insecticidal Cry toxins. However, the mechanism preceding pore formation by Cry4Aa, including receptor recognition, remains to be elucidated. The present study examined the potential involvement of sugar moieties in the Cry4Aa mode of action, with a particular focus on the process of pore formation. Cry4Aa and the sugar *N*-acetylgalactosamine exhibited considerably increased binding affinity. Furthermore, *N*-acetylgalactosamine treatment significantly enhanced the toxicity of Cry4Aa against *Culex pipiens* mosquito larvae. In the presence of *N*-acetylgalactosamine, the binding of Cry4Aa to phosphatidylcholine liposomes increased by 1.4-fold. The presence of this sugar did not affect the single-channel conductance of Cry4Aa pores formed in artificial lipid bilayers; however, it did appear to increase the number of Cry4Aa pores per designated area. *In silico* molecular docking analysis revealed promising binding sites within the cavity, surrounded by the Cry4Aa domains. These results suggest that the interaction of Cry4Aa with *N*-acetylgalactosamine moieties is a vital step in the process of pore formation. This interaction may induce a conformational change in the Cry4Aa molecule that facilitates its insertion into the target membrane.

**IMPORTANCE:**

Cry toxins produced by the soil bacterium *Bacillus thuringiensis* are used as an environmentally friendly biopesticide. Cry toxins are generally considered pore-forming toxins; however, the mechanism preceding pore formation appears to be complex and is not yet fully elucidated. This study examined the interaction between mosquito-larvicidal Cry4Aa toxin and sugar moieties, determining considerably higher binding affinity with *N*-acetylgalactosamine. Furthermore, treatment with this sugar significantly increased the toxicity of Cry4Aa toward *Culex pipiens* mosquito larvae. Subsequent analyses revealed that treatment with this sugar increases the binding of Cry4Aa to phosphatidylcholine bilayers, thereby facilitating pore formation. The findings of this study enhance our understanding of the mechanism underlying the insecticidal activity of Cry toxins.

## INTRODUCTION

The gram-positive, spore-forming bacterium *Bacillus thuringiensis* serovar *israelensis* (Bti) exhibits strong toxicity against larvae of various medically important mosquito genera, including *Aedes*, *Anopheles*, and *Culex* (1, 2). Bti therefore has a long history of use as a mosquito-control agent (3, 4). The mosquito-larvicidal activity of Bti derives from three Cry toxins (Cry4Aa, Cry4Ba, and Cry11Aa) and the Cyt1Aa toxin (5, 6). These toxins are primarily synthesized as inactive protoxins within the parasporal crystalline inclusion. Upon ingestion by susceptible mosquito larvae, the crystalline inclusions are solubilized in the alkaline environment of the midgut lumen, and the contents are subsequently processed by gut proteases, yielding active toxins (7). Bti Cry toxins demonstrate considerable toxicity against a range of mosquito larvae. According to the colloid-osmotic lysis model, after complex interaction with specific receptors, the active toxins form pores that allow ions and water molecules to enter target cells, which disrupts the membrane potential and leads to swelling and the eventual death of the target cell due to lysis (8, 9). Cyt1Aa, by contrast, exhibits low mosquito-larvicidal activity but synergizes with other Bti Cry toxins (10–14). Interestingly, synergistic toxicity has also been observed between Cry toxins (2, 15–17). The observed synergistic effect of Bti toxins appears to be advantageous for mosquito control; however, the underlying mechanism remains to be fully elucidated.

Three-dimensional structure models of Cry4Aa, Cry4Ba, and Cry11Aa have been constructed using X-ray crystallography (18–20), which revealed that the toxins have a similar architecture consisting of three domains (I, II, and III). In general, the N-terminal domain (I) consists of a bundle of seven α-helices thought to form the transmembrane pore. The central domain (II), which consists of three antiparallel β-sheets, functions as a receptor-binding domain. The C-terminal domain (III) consists of antiparallel β-sheets that form a β-sandwich fold with a jellyroll topology. Domain III is assumed to play a role in controlling the structural integrity and/or receptor binding of the toxin (5). By contrast, Cyt1Aa has a single domain composed of β-sheets in the center and surrounded by two α-helical layers (21). Cyt1Aa reportedly interacts directly with saturated membrane lipids, including phosphatidylcholine (PC), phosphatidylethanolamine, and sphingomyelin (22).

It is noteworthy that, despite their structural similarity, Bti Cry toxins (particularly Cry4Aa) appear to have diverging modes of action. Cry4Aa reportedly forms cation-selective channel pores in planar lipid bilayers (23). Our previous mutational analyses revealed an apparent correlation between mosquito-larvicidal activity and the ion permeability of Cry4Aa pores (24). These observations suggest that the toxicity of Cry4Aa is primarily related to pore formation, as is the case with many other Cry toxins. However, the mechanism underlying the receptor recognition process, a major event preceding pore formation, appears to be complex and has not yet been fully elucidated for Cry4Aa. For example, the domain II loops, particularly loops 1, 2, and 3, are reportedly involved in the toxin-receptor interaction for many Cry toxins (25–29). However, substitution of the amino acid residues in these loops of Cry4Aa does not significantly diminish the mosquito-larvicidal activity of the toxin (30). We therefore hypothesized that multiple subsites that function cooperatively in receptor binding are spread throughout domains II and/or III of Cry4Aa.

Cry4Aa domain III shares similar structural features with the cellulose-binding domain of a protein from *Cellulomonas fimi* (31) and *Clostridium thermocellum* xylanase (32). This structural similarity suggests that a sugar moiety may play a role in the binding of Cry4Aa to its receptor. Indeed, previous studies have reported the involvement of domain III in the insecticidal activity of the lepidopteran-specific toxins Cry1Ac (33–36) and Cry1C (36). Cry1Ac was shown to bind its receptor, aminopeptidase N (APN), through domain III, which recognizes *N*-acetylgalactosamine (GalNAc) moieties (33).

The present study examined the interaction between Cry4Aa and sugar moieties. To investigate the potential involvement of sugar moieties in the mechanism of action of Cry4Aa, the binding affinity between Cry4Aa and selected sugars (GalNAc, *N*-acetylglucosamine [GlcNAc], galactose, mannose, and fucose) was determined. The effect of these sugars on mosquito-larvicidal activity was then estimated using a bioassay with *Culex pipiens* mosquito larvae. In addition, electrophysiologic measurements using artificial lipid bilayers were conducted to assess the relationship between sugar binding and associated mosquito-larvicidal activity. Furthermore, potential GalNAc binding sites on the surface of Cry4Aa molecules were identified using a protein-ligand bind docking tool.

## MATERIALS AND METHODS

### Preparation of recombinant Cry4Aa

Cry4Aa active toxin fragment (G^58^–Q^695^) fused with glutathione *S*-transferase (GST-Cry4Aa) was expressed in *Escherichia coli* BL21, as described previously (37). The *E. coli* cells were cultured in TB medium containing ampicillin (100 μg/mL) until the OD_600_ reached 0.5–0.7. Expression of recombinant Cry4Aa was then induced by incubating the cells in 0.1 mM isopropyl-β-D-thiogalactopyranoside at 30°C for 4 h. Following disruption of the cells by sonication, recombinant Cry4Aa was purified using glutathione-Sepharose 4B resin (GE Healthcare Bio-Sciences AB, Uppsala, Sweden) according to the manufacturer’s instructions. The active toxin fragment of Cry4Aa was cleaved from GST-Cry4Aa bound to the resin using thrombin (Cytiva, Tokyo, Japan), as described previously (23). Protein concentration was estimated using a protein assay kit (Bio-Rad Laboratories, Inc., Hercules, CA, USA) with bovine serum albumin as the standard.

### Quartz crystal microbalance assay

Interactions between Cry4Aa and sugars were analyzed using a Single Q 0500 QCM device (As One, Osaka, Japan). A gold sensor chip was coated with active Cry4Aa toxin (7 μg/mL) and blocked with 4% (wt/vol) Blocking One reagent (Nacalai Tesque, Kyoto, Japan) in PBS. Next, one of the five test sugars (GalNAc, GlcNAc, galactose, mannose, or fucose) was applied to the gold sensor chip. The dissociation constant (*K*_*D*_) was obtained from a graph of the concentration of applied sugar versus the mass increase of the polypeptide on the sensor surface (in Hertz), as determined from the QCM sensorgram. Each experiment was performed independently three times to obtain the kinetic parameters. Q-up analysis software, version 1.02 (Scinics, Tokyo, Japan), was used for curve fitting, construction of Hanes-Woolf plots, and calculation of *K*_*D*_ values, as described previously (38).

### Measurement of mosquito-larvicidal activity

The mosquito-larvicidal activity of Cry4Aa was analyzed using bioassays with *C. pipiens* mosquito larvae (third instar). Mosquito larvae were reared from eggs kindly supplied by the Research and Development Laboratory, Dainihon Jochugiku Co., Ltd. (Osaka, Japan). Bioassays were performed as described previously (30, 39), with some modifications. Briefly, 40 μg of purified GST-Cry4Aa was adsorbed onto 2 mg of latex beads (0.8-μm diameter, Sigma-Aldrich Corp., St. Louis, MO, USA) for 1 h at room temperature and then administered to the mosquito larvae as a diet. GST-Cry4Aa was pre-incubated in a 100 mM solution of one of the sugars (GalNAc, GlcNAc, galactose, mannose, or fucose) for 30 min. The GST-Cry4Aa solution containing sugars was used directly for adsorption onto latex beads and then administered to the mosquito larvae. Bioassays were carried out in 96-well microtiter plates with a single larva per well and 48 larvae per each concentration per assay. Mortality was recorded 48 h after toxin administration, and PROBIT analysis was used to determine the 50% lethal concentration (LC_50_) and 95% confidence limits (40, 41). All bioassay experiments were repeated three times.

### Detection of Cry4Aa binding to PC liposomes

PC liposomes were generated as described previously (42, 43) using magnetite particles (200 μm diameter). Five milligrams of PC was dissolved in 1 mL of chloroform, and then a thin film of PC was generated by evaporation of the chloroform. The film was suspended in 1 mL of PBS containing 1 mg of magnetite particles. The magnetite particles were kindly supplied by Toda Kogyo Corp. (Hiroshima, Japan) and pretreated with Blocking One solution (Nacalai Tesque). PC liposomes were pelleted using a magnet and then incubated in PBS containing active Cry4Aa toxin fragment (25 μg/mL) with or without 100 mM GalNAc. After 30 min of incubation at room temperature, the PC liposomes were pelleted using a magnet and washed twice with PBS. The pelleted PC liposomes were suspended in 100 μL of PBS and then spotted onto a polyvinylidene difluoride membrane using a vacuum-based dot blot apparatus (Bio-Dot microfiltration apparatus; Bio-Rad Laboratories, Inc.). Cry4Aa bound to PC liposomes was detected using anti-Cry4Aa rabbit antiserum followed by anti-rabbit IgG goat antibodies conjugated with horseradish peroxidase (American Qualex Antibodies, San Clemente, CA) and visualized using ECL Western blotting substrates (Promega Corp., Madison, WI). Binding of Cry4Aa to PC liposomes was estimated by densitometric scanning using a cooled CCD camera system (EZ-Capture; ATTO Corp., Tokyo, Japan).

### Electrophysiologic analysis

Electrophysiologic analyses of Cry4Aa pores were performed using agarose gel-supported lipid bilayers constructed as previously described (44). Briefly, the electrophysiologic apparatus consisted of two chambers (*cis* and *trans*), such that the voltage in the *cis* chamber was connected to a patch-clamp amplifier via a membrane potential defined by an Ag/AgCl electrode. The *cis* chamber consisted of a generic gel-loading tip filled with recording solution (150 mM KCl, 10 mM Tris-HCl [pH 8.0]) containing 1% agarose. The agarose was allowed to solidify, with a small amount protruding (1 μL) from the tip. By contrast, a generic 0.6-mL microtube was used as the *trans* chamber. The microtube contained activated Cry4Aa toxin (1.0, 1.5, or 2.5 μg/mL), with or without GalNAc (5 mM) in recording solution, which was overlaid with PC solution (20 mg/mL in *n*-decane). Lipid bilayers were constructed at the interface between the lipid solution and the recording solution in the *trans* chamber. Subsequent to insertion of the *cis* chamber into the lipid solution and the placement of a 1% agarose gel protrusion at the interface, the lipid bilayers usually appeared as circular forms with a diameter of approximately 0.3 mm (44).

After detection of a current spike indicative of toxin pore formation in the lipid bilayers, the current was recorded between −70 and +70 mV. To ensure the reliability of the results, the measurement was repeated 15 times using the same toxin sample in the presence and absence of GalNAc. Data were analyzed using PCLAMP software, ver. 11.1 (Axon Instruments, Foster City, CA, USA). In each case, detected current was plotted against the corresponding applied voltage to generate current–voltage relationship graphs. Channel conductance was determined from the slope of the current–voltage relationship.

### *In silico* molecular docking analysis

Potential binding sites for GalNAc on the surface of Cry4Aa molecules were identified through a search aided by the protein-ligand blind docking tool from the CB-Dock2 server (https://cadd.labshare.cn/cb-dock2/php/index.php). Cavity-detection-guided molecular docking (45, 46) was performed using structure data files for Cry4Aa (PDB 2C9K) and GalNAc (PubChem CID: 35717). Upon prediction of potential cavities for GalNAc binding, candidate sites were sorted based on binding affinity. The binding affinity of GalNAc for each predicted cavity in Cry4Aa was evaluated based on the Vina score (47). Furthermore, the analysis for two additional Bti mosquito-larvicidal toxins, Cry4Ba (PDB 1W99) and Cry11Aa (PDB 7QX4), was also conducted in this study.

## RESULTS

### Preparation of recombinant Cry4Aa

**Fig 1:**
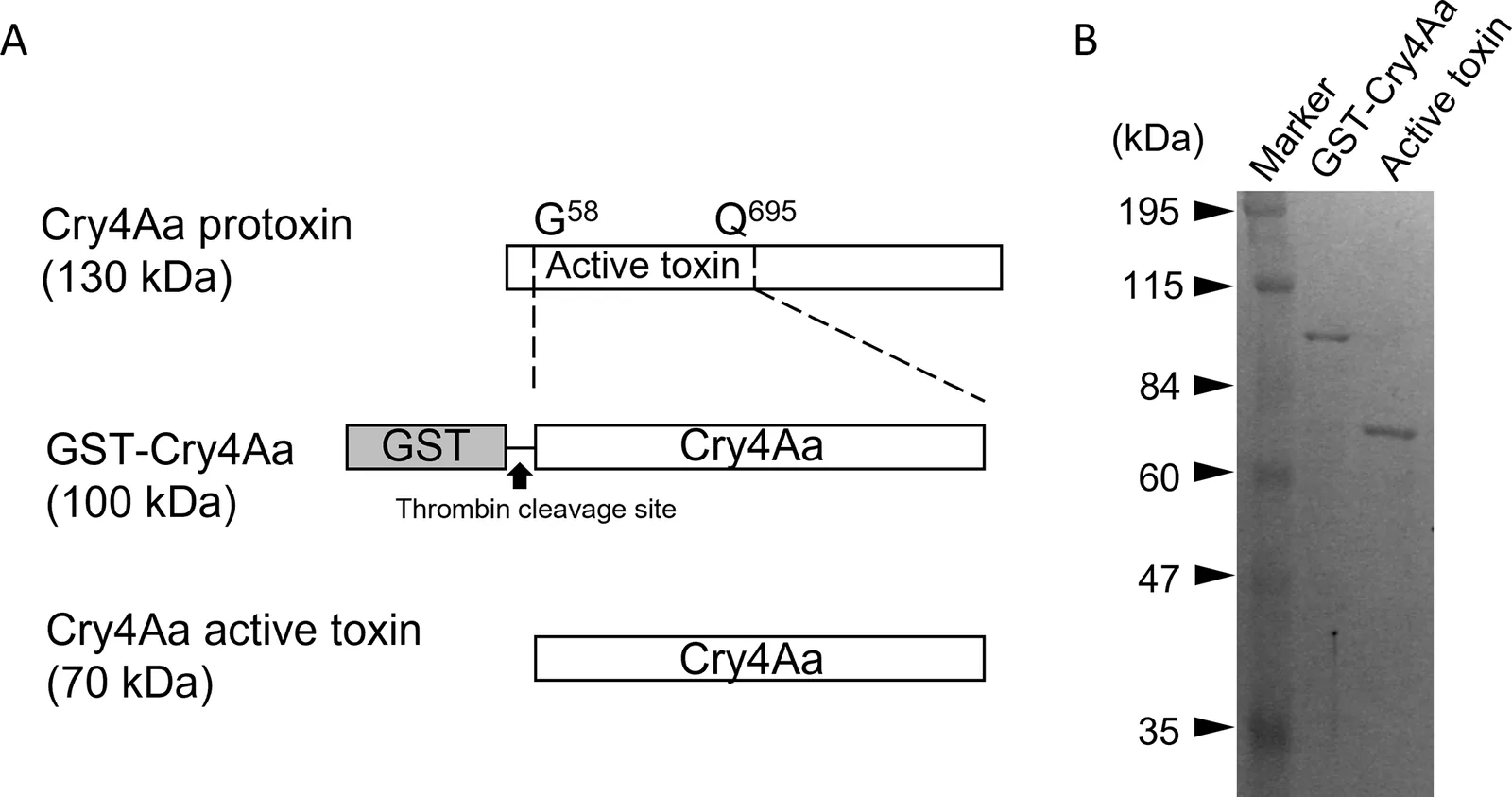
Preparation of recombinant Cry4Aa. (**A**) Schematic illustration of the structure of recombinant Cry4Aa. In this study, active Cry4Aa toxin (G58–Q695) was expressed as a GST fusion (GST-Cry4Aa). The active toxin fragment of Cry4Aa was cleaved from GST-Cry4Aa using thrombin. (**B**) SDS-PAGE (10%) analysis of purified proteins. A total of 0.5 μg of purified protein was applied in each lane.

Recombinant Cry4Aa was successfully expressed and purified. The purified proteins were subjected to sodium dodecyl sulfate–polyacrylamide gel electrophoresis (SDS-PAGE), which revealed that GST-Cry4Aa (approximately 100 kDa) and the active toxin fragment of Cry4Aa (approximately 70 kDa) were highly purified (Fig. 1).

### Interaction between Cry4Aa and various sugars

**Fig 2:**
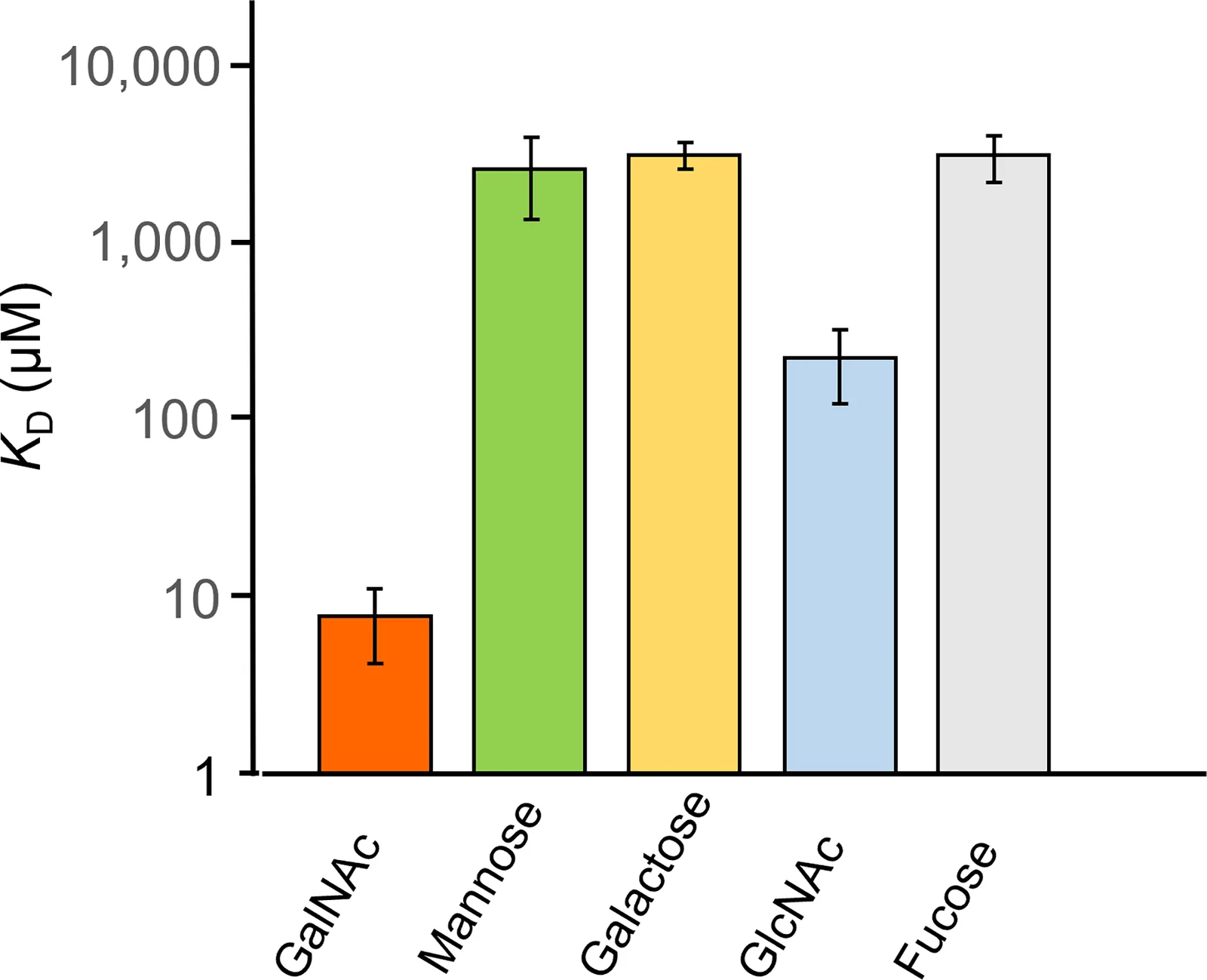
Binding affinity (*K*_*D*_) of Cry4Aa for various sugars. A gold sensor chip was coated with active Cry4Aa toxin (7 μg/mL), and then one of five sugars (GalNAc, GlcNAc, galactose, mannose, or fucose) was applied upon blocking and washing of the chip. Each experiment was repeated three times, and average values with standard deviation are shown.

In this study, we initially analyzed the binding affinity between Cry4Aa and five selected sugars (GalNAc, GlcNAc, galactose, mannose, and fucose) using a quartz crystal microbalance (QCM) device. Consequently, a substantially higher binding affinity was observed for GalNAc, with a *K*_*D*_ value of 8 ± 3 µM, followed by GlcNAc, with a *K*_*D*_ value of 231 ± 104 µM (Fig. 2). The remaining tested sugars (galactose, mannose, and fucose) exhibited low binding affinity, with *K*_*D*_ values > 2 mM (Fig. 2).

### Effect of sugar treatment on the mosquito-larvicidal activity of Cry4Aa

**Fig 3:**
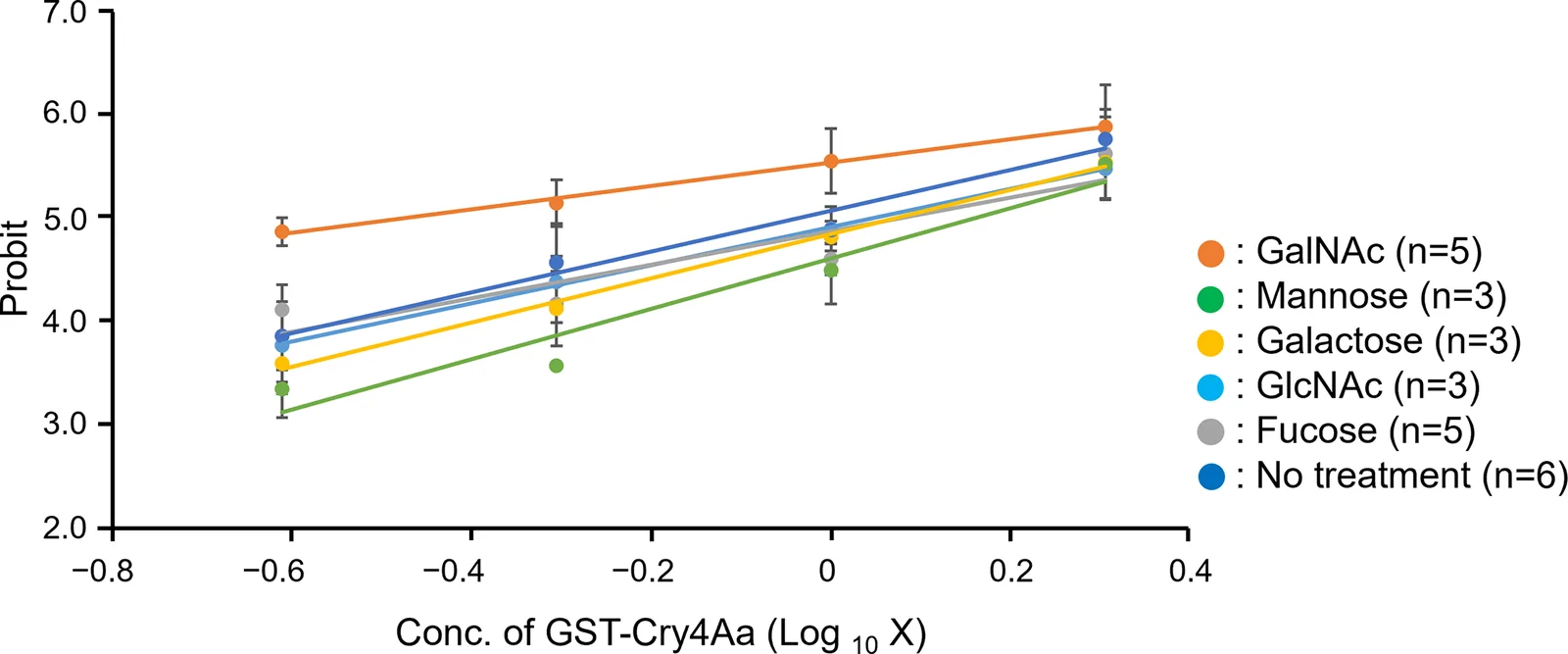
Mosquito-larvicidal activity of GST-Cry4Aa. GST-Cry4Aa was pretreated with designated sugars. The mortality rate of mosquito larvae was recorded 48 h after administration, and log (dose) vs. probit plot is shown. Bioassays were repeated more than three times, and average values with standard deviation are shown. X: concentration of GST-Cry4Aa (μg/mL).

The effect of sugar treatment on the toxicity of Cry4Aa was investigated further using a bioassay with *C. pipiens* mosquito larvae. The LC_50_ value (95% confidence limit) of GST-Cry4Aa without sugar treatment was 1.15 (1.09–1.23) µg/mL (Fig. 3), similar to that reported previously (30, 39).

The highest rate of larval mortality was observed with administration of GalNAc-treated GST-Cry4Aa, with a corresponding LC_50_ value of 0.36 (0.32–0.40) µg/mL, more than three times higher than that of GST-Cry4Aa without sugar treatment (Fig. 3). The LC_50_ value of GlcNAc-treated GST-Cry4Aa was 0.95 (0.91–1.00) µg/mL, indicating that the toxicity of the sugar-treated toxin was slightly higher than that of untreated GST-Cry4Aa. The toxicity of GST-Cry4Aa treated with galactose, mannose, or fucose was similar to that of untreated GST-Cry4Aa, with LC_50_ values of 1.21 (1.15–1.27), 1.44 (1.38–1.52), and 1.27 (1.19–1.37) µg/mL, respectively (Fig. 3). On the other hand, no detectable mosquito-larvicidal activity was observed with the administration of GSTs, whether treated or untreated with sugars, at least within the tested concentrations (up to 2 μg/mL). These observations indicate that specific interaction with GalNAc enhances the mosquito-larvicidal activity of Cry4Aa.

### Effect of GalNAc treatment on Cry4Aa membrane binding

**Fig 4:**
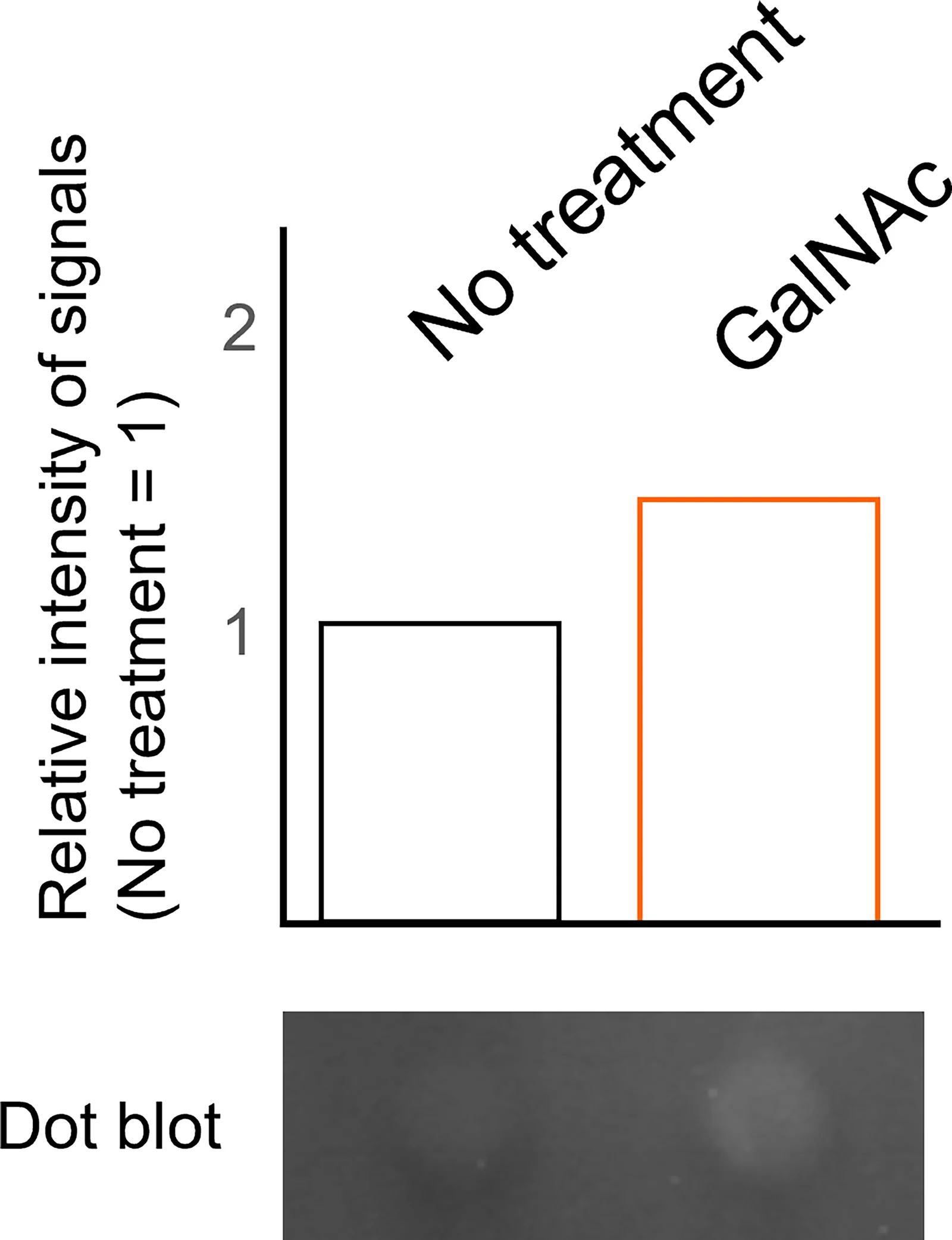
Dot blot analysis. Active Cry4Aa toxin was incubated with PC liposomes containing magnetic particles, and Cry4Aa bound to the liposomes was analyzed by dot blot using anti-Cry4Aa antiserum. Binding of Cry4Aa treated with GalNAc is expressed as a ratio compared to binding of Cry4Aa without sugar treatment.

In order to assess the effect of GalNAc treatment on Cry4Aa membrane binding, active Cry4Aa toxin was incubated with PC liposomes. Bound toxin was then detected by dot blotting using anti-Cry4Aa antiserum. Despite the absence of Cry4Aa receptors in PC liposomes, a slight but discernible binding signal was detected (Fig. 4). Moreover, pretreatment with GalNAc increased the amount of Cry4Aa that bound to PC liposomes by 1.4-fold (Fig. 4). We assumed that the Cry4Aa detected in this assay was toxin integrated into the liposomes rather than toxin attached to the liposome surface. We hypothesized that interaction with GalNAc induced a conformational change in the Cry4Aa molecule that facilitated its insertion into the PC liposomes.

### Effect of GalNAc treatment on Cry4Aa pore formation

**Fig 5:**
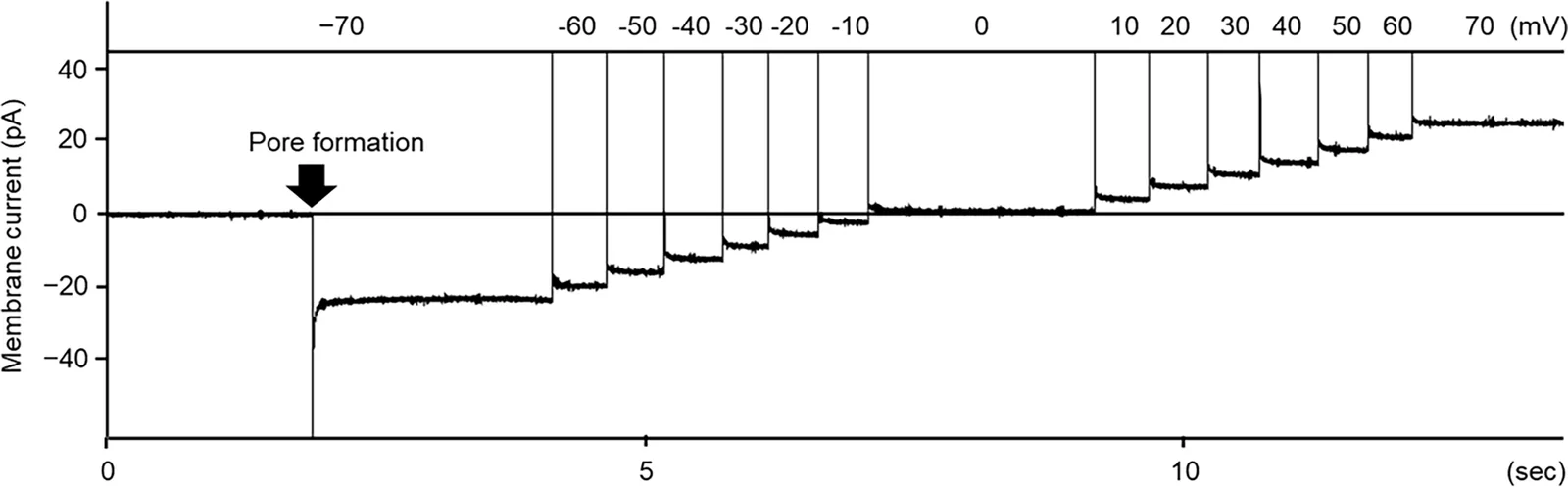
Representative segment of a typical current trace (Cry4Aa, without sugar treatment). The arrow shows the current spike, indicating pore formation.

The present study investigated the impact of GalNAc treatment on Cry4Aa pore formation using an electrophysiologic approach. Pore formation by Cry4Aa was initially analyzed in the absence of GalNAc. The formation of Cry4Aa pores was facilitated by applying a voltage of −70 mV. Subsequently, a current spike indicative of toxin pore formation was detected and remained in a stable open state for several minutes (Fig. 5).

**Fig 6:**
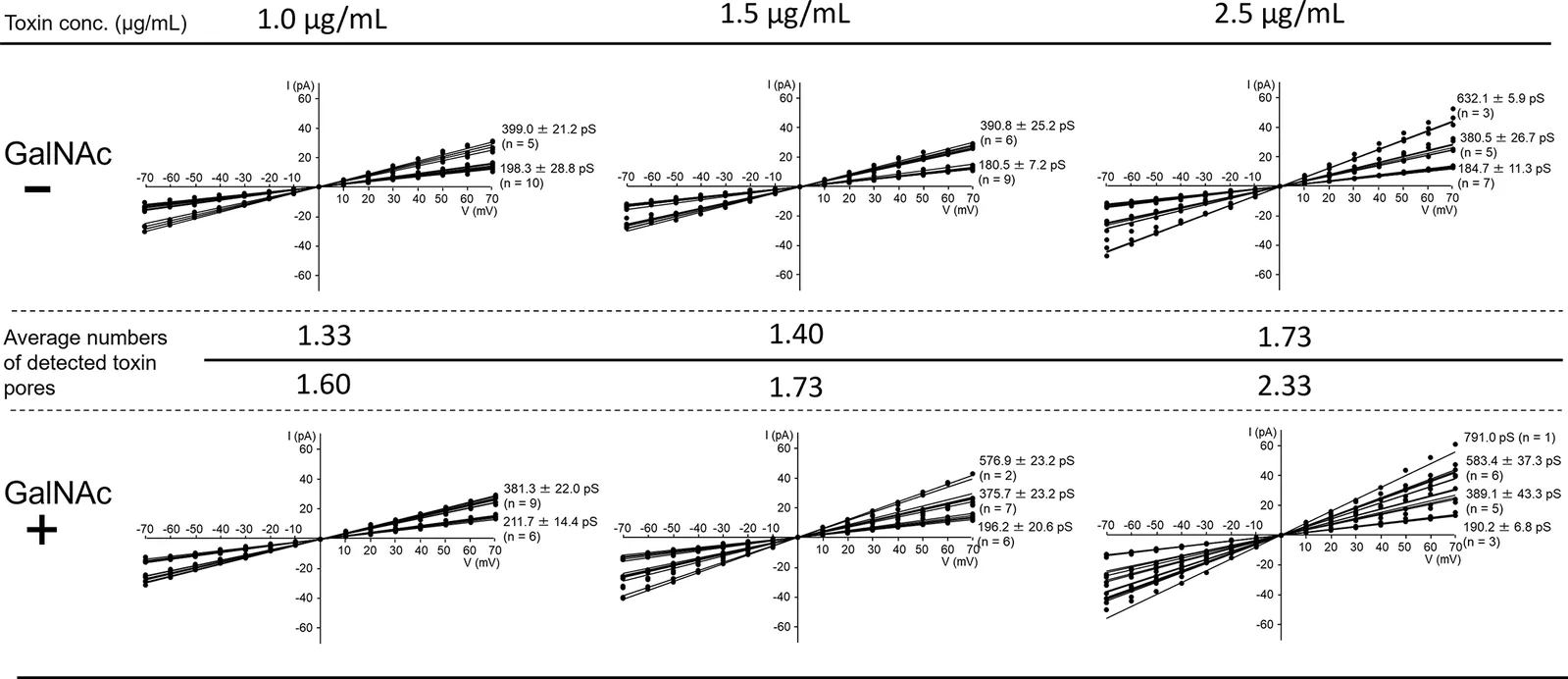
Current–voltage relationships for Cry4Aa channel pores in the presence or absence of 5 mM GalNAc. Channel currents, recorded in symmetric 150 mM KCl solution, were plotted against applied voltage. Measurements were repeated 15 times, and the average numbers of detected toxin pores are shown.

The current, ranging from −70 to +70 mV, was plotted against the corresponding applied voltage, thereby generating current–voltage relationships, which were generally linear. However, lines exhibiting multiple conductance levels were observed, especially in measurements at higher toxin concentrations (Fig. 6). Conductance with higher levels was attributed to the presence of multiple toxin pores, as these were almost universally in multiples of the lowest conductance (Fig. 6). Thus, the single-channel conductance of Cry4Aa pores was 188.1 ± 32.3 pS (*n* = 45) in symmetrical buffer containing 150 mM KCl. This value was similar to the previously established conductance of approximately 187 pS for Cry4Aa pores (23, 44). It is noteworthy that the average number of Cry4Aa pores per designated area (~0.3 mm diameter circle) appeared to correlate with toxin concentration, with 1.33 pores formed in 1.0 μg/mL toxin, 1.40 pores in 1.5 μg/mL toxin, and 1.73 pores in 2.5 μg/mL toxin (Fig. 6). This observation suggests that the conductance value may serve as an effective indicator of toxin pore formation efficiency.

The impact of GalNAc on the Cry4Aa pore formation was analyzed using a recording solution supplemented with 5 mM GalNAc in a *trans* chamber. Cry4Aa pores demonstrated a profile very similar to that obtained in measurements using GalNAc-free recording solution (Fig. 6). The single-channel conductance of Cry4Aa pores was 195.0 ± 15.1 pS (*n* = 45). Notably, this outcome was similar to results obtained in measurements conducted in the absence of GalNAc, indicating that GalNAc treatment did not affect the size of Cry4Aa pores. By contrast, the average number of Cry4Aa pores per designated area appeared to increase with increasing toxin concentration, with 1.60 pores in 1.0 μg/mL toxin, 1.73 pores in 1.5 μg/mL toxin, and 2.33 pores in 2.5 μg/mL toxin (Fig. 6). This result appeared to correlate well with that observed in the analysis of Cry4Aa binding to PC liposomes (Fig. 4). GalNAc treatment is thought to facilitate the insertion of Cry4Aa molecules into the PC membrane, thereby increasing the average number of toxin pores per designated area.

### *In silico* molecular docking analyses

**Fig 7:**
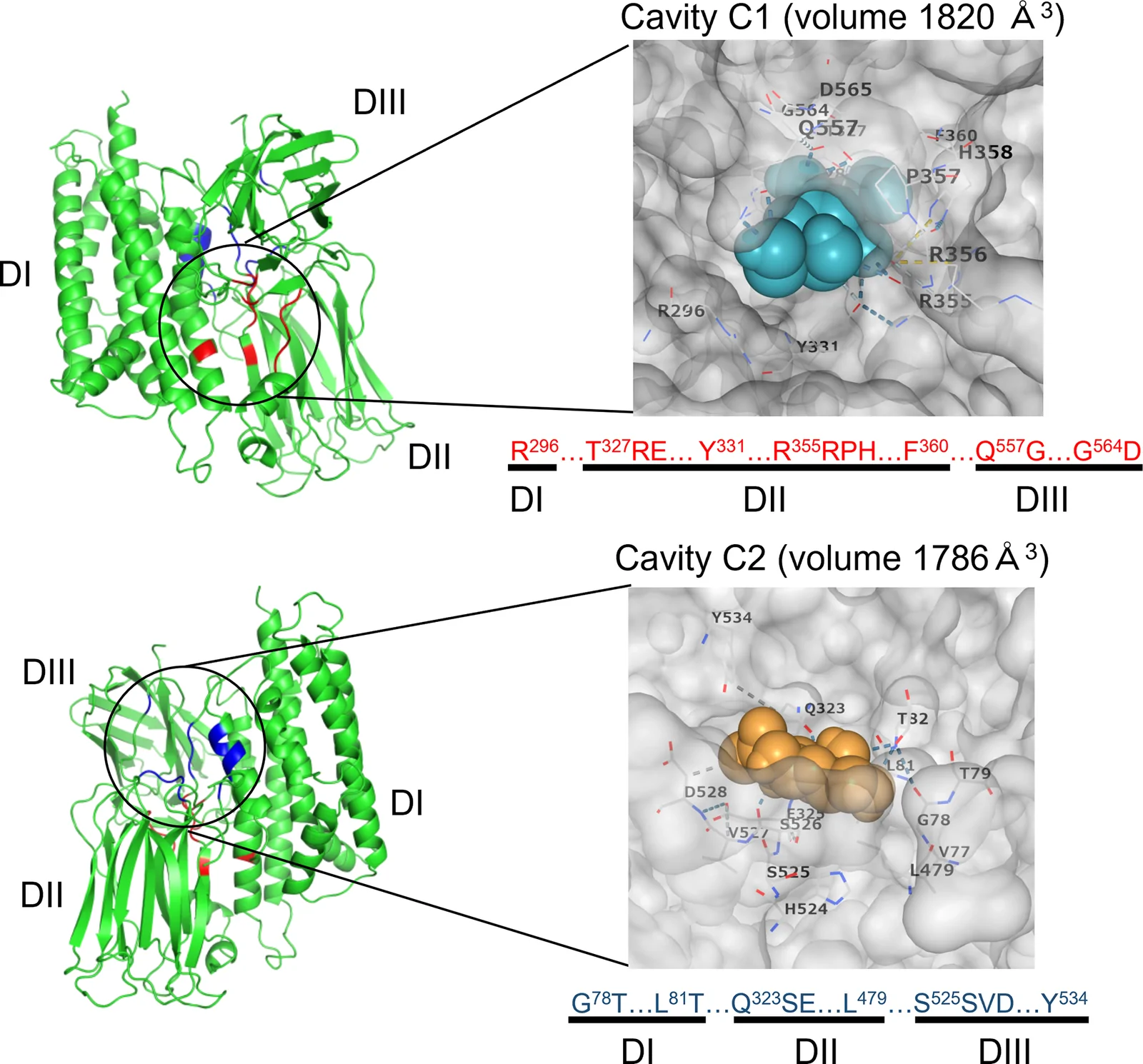
Promising GalNAc binding sites (cavities C1 and C2) identified on the surface of Cry4Aa molecules. The search was aided by the protein-ligand blind docking tool from the CB-Dock2 server (https://cadd.labshare.cn/cb-dock2/php/index.php). Analyses were performed using structure data files for Cry4Aa (PDB 2C9K) and GalNAc (PubChem CID: 35717). Amino acid residues forming cavities C1 and C2 are indicated in red and blue, respectively. GalNAc bound to the cavity is indicated using a space-filling model.

**Fig 8:**
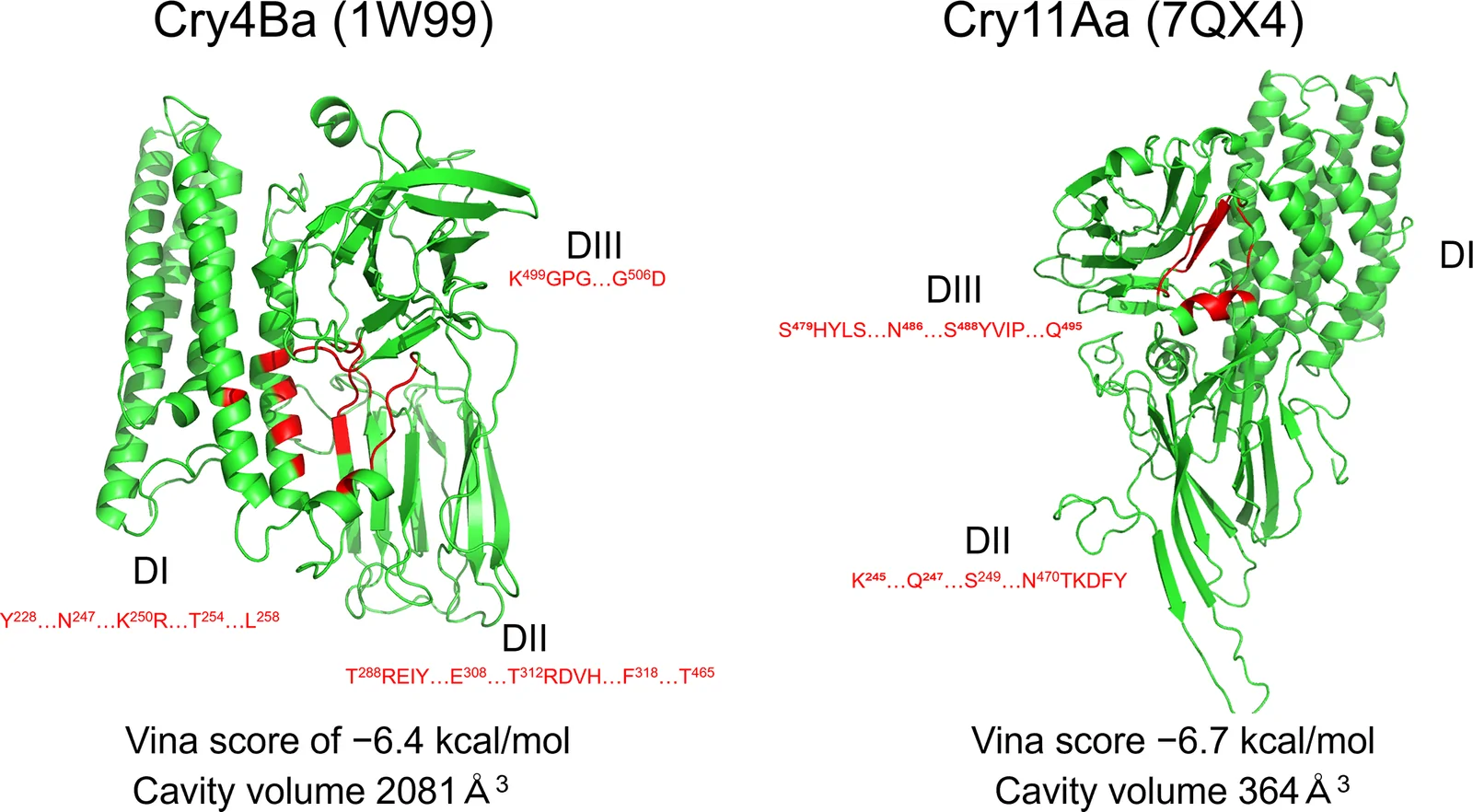
Promising GalNAc binding sites identified on the surface of Cry4Ba and Cry11Aa molecules. The search was aided by the protein-ligand blind docking tool from the CB-Dock2 server (https://cadd.labshare.cn/cb-dock2/php/index.php). Analyses were performed using structure data files for Cry4Ba (PDB 1W99), Cry11Aa (PDB 7QX4), and GalNAc (PubChem CID: 35717). Amino acid residues forming cavities are indicated in red.

Potential GalNAc binding sites on the surface of Cry4Aa molecules were identified using a protein-ligand blind docking tool. The most promising candidate sites were located in large cavities surrounded by the three Cry4Aa domains (I, II, and III). The cavities in which GalNAc bound with the lowest energy were tentatively designated cavities C1 and C2 (Fig. 7). Cavity C1 consisted of R^296^ of domain I, T^327^RE, Y^331^, R^355^RPH, and F^360^ of domain II, and Q^557^G and G^564^D of domain III, with an estimated cavity volume of 1,820 Å^3^ and Vina score of −6.6 kcal/mol for GalNAc binding (Fig. 7). Cavity C2 was located on the opposite side of C1 of the Cry4Aa molecule (Fig. 7) and consisted of G^78^T and L^81^T of domain I, Q^323^SE and L^479^ of domain II, and S^525^SVD and Y^534^ of domain III, with an estimated cavity volume of 1,786 Å^3^ and Vina score of −5.1 kcal/mol for GalNAc binding (Fig. 7). The most promising GalNAc binding site identified in Cry4Aa domain III was a cavity (volume = 217 Å^3^) consisting of G^596^S, T^600^, and V^661^YTNT, with a Vina score of −4.3 kcal/mol. Interestingly, the molecular docking analysis revealed one promising GalNAc binding site analogous to those observed in Cry4Aa cavities C1 and C2 in another Bti mosquito-larvicidal Cry4Ba. This site was constituted of Y^228^, N^247^, K^250^R, T^254^, and L^258^ of domain I, T^288^REIY, E^308^, T^312^RDVH, F^318^, and T^465^ of domain II, and K^499^GPG and G^506^D of domain III, with an estimated cavity volume of 2,081 Å^3^ and Vina score of −6.4 kcal/mol for GalNAc binding (Fig. 8). In addition, the analysis also indicated the presence of multiple minor candidates within the various cavities of Cry4Ba molecules. The most promising GalNAc binding site identified in Cry11Aa (Vina score = −6.7 kcal/mol) was located in the smaller cavity (cavity volume = 364 Å^3^), comprising K²⁴⁵, Q²⁴⁷, S^249^, N^470^TKDFY of domain II, and S⁴⁷⁹HYLS, N⁴⁸⁶, S⁴⁸⁸YVIP, and Q⁴⁹⁵ of domain III (Fig. 8). Thus, GalNAc binding sites were found not only in Cry4Aa but also in Cry4Ba and Cry11Aa, indicating the possibility of interaction with a GalNAc moiety. Nevertheless, the dimensions of the cavities and the location within the toxin molecule demonstrate variability, thereby suggesting a discrepancy in the manner of interaction with the GalNAc moiety. The predicted GalNAc binding sites identified in the toxin molecules will require experimental validation via site-directed mutagenesis, followed by assays including binding with sugar moieties and toxicity, to elucidate their functional significance in the future.

## DISCUSSION

Multiple studies have reported that Cry toxin domain II has structural similarities to various carbohydrate-binding proteins, including lectin jacalin, lectin Mpa, and vitellin (6, 41, 43, 48, 49). Cry toxin domain III also reportedly shares structural similarity with other carbohydrate-binding proteins, such as the cellulose-binding domain of 1,4-β-glucanase C, β-glucuronidase, β-galactosidase, galactose oxidase, sialidase, and xylanase U (50). Similar structural features have also been identified in the domains II and III of Cry4Aa (19). These similarities with carbohydrate-binding proteins suggest that sugar moieties play a role in the mode of action of Cry toxins. Consequently, the present study examined the potential involvement of sugar moieties in the mode of action of Cry4Aa, with a particular focus on the pore formation process. Binding assays employing a QCM device revealed a considerably higher binding affinity between Cry4Aa and GalNAc relative to other sugars (Fig. 2). Furthermore, bioassays using *C. pipiens* larvae demonstrated that treatment with GalNAc enhances the mosquito-larvicidal activity of Cry4Aa (Fig. 3). Thus, the interaction of Cry4Aa molecules with the GalNAc moiety appears to play a role in the toxin’s mode of action.

Cry4Aa is a pore-forming toxin (23), and the findings of our prior investigations indicate that the toxicity of Cry4Aa is predominantly associated with the characteristics of toxin pores (23, 24). Thus, we hypothesized that the binding of toxin molecules to GalNAc facilitates one or more steps involved in pore formation by Cry4Aa, such as membrane insertion. Indeed, we found that GalNAc treatment increased the binding of Cry4Aa to PC liposomes by 1.4-fold (Fig. 4). Moreover, GalNAc treatment did not affect the single-channel conductance of Cry4Aa pores formed in artificial lipid bilayers; however, GalNAc treatment did appear to increase the number of Cry4Aa pores per designated area (Fig. 6). These findings suggest that the interaction of the toxin with the GalNAc moiety induces a conformational change in the Cry4Aa molecule that facilitates pore formation, thereby enhancing the toxin’s insecticidal activity. Conformational changes of the toxin molecule prior to membrane insertion have been observed in Cry1Ac and Cry1Ab (51–53). Additionally, recent research has documented membrane-induced conformational changes for Cry4Ba monomers, leading to the assembly of a pre-pore trimer structure (54). In this study, direct evidence indicating analogous structural changes has yet to be observed in the Cry4Aa molecule upon interaction with GalNAc. However, the single-channel analysis employed in this study is highly sensitive and capable of detecting a single toxin pore formed in lipid bilayers. This high level of capability may have facilitated the identification of alterations in the Cry4Aa molecule.

Interestingly, an effect of GalNAc on the insecticidal activity of other Cry toxins has also been reported. Lepidopteran-specific Cry1Ac reportedly binds its receptor, APN, via domain III, which recognizes GalNAc moieties (33, 55). Consequently, the presence of GalNAc inhibits the binding of Cry1Ac to APN, thereby decreasing its toxicity. Thus, the effect of GalNAc binding on Cry1Ac toxicity differs markedly from the effect of binding on Cry4Aa toxicity. The divergent effect of GalNAc binding on toxicity might be explained by a difference in the GalNAc binding site in the Cry4Aa and Cry1Ac toxin molecules. A cavity-detection-guided molecular docking analysis identified two promising candidate sites in Cry4Aa (cavities C1 and C2) for GalNAc binding. In accordance with our hypothesis, these cavities were located between the three domains (I, II, and III) rather than within domain III (Fig. 7). If these candidate sites are indeed involved in binding to the GalNAc moiety, it is plausible that this binding induces a conformational change in the Cry4Aa molecule that facilitates pore formation by the toxin. The docking analysis also revealed potential GalNAc binding sites in Cry4Ba and Cry11Aa, as well as in Cry4Aa (Fig. 8). The potential GalNAc binding sites within the toxin molecule appeared to vary among toxins, suggesting the possibility of variations in the mode of action of Bti Cry toxins.

In the widely accepted pore formation model, Cry toxin molecules activated in the midgut of susceptible insect larvae bind with low affinity to glycosylphosphatidylinositol (GPI)-anchored receptors such as APN and/or alkaline phosphatase (ALP). This binding is thought to promote localization and concentration of the Cry toxin. The Cry toxin molecules then bind to the cadherin receptor, which facilitates proteolytic cleavage of domain I α1 and induces the formation of a pre-pore oligomer. This Cry toxin oligomer exhibits enhanced binding affinity for GPI-anchored receptors, which induces a conformational change in the Cry toxin that leads to membrane insertion, followed by pore formation and cell lysis (7). Considering our findings in relation to this model, interaction with a GalNAc moiety on the receptor may be one of the final steps prior to pore formation, and this interaction could induce a conformational change in the Cry4Aa molecule that facilitates membrane insertion of the α-helix of domain I.

Research has demonstrated the presence of multiple receptors such as APNs, ALPs, and cadherins in mosquitoes for Cry4Ba and Cry11Aa (56, 57). The receptor proteins for Cry4Ba and Cry11Aa appeared to be glycosylated; however, the role of sugar moieties in toxin-receptor binding remains to be fully elucidated (56). On the other hand, the receptors for Cry4Aa remain to be elucidated. The presence of multiple binding proteins with a lower molecular mass (18–30 kDa) has been detected in brush border membrane vesicles extracted from *C. pipiens* larvae (58). In addition, two GPI-anchored ALP isoforms, *Cq*-mALP1263 and *Cq*-mALP1264, were identified in *C. quinquefasciatus* larvae (59). These are structurally similar to those of *A. aegypti* ALP, which is identified as Cry4Ba receptor, and their expression enhances susceptibility against Cry4Aa in *Spodoptera frugiperda* (Sf9) cultured cells.

It is reasonable to speculate that the presence of the receptors varies among different species of mosquitoes. Cry4Aa has been demonstrated to be particularly toxic to *Aedes* and *Culex* larvae, while exhibiting slightly reduced toxicity against *Anopheles* larvae (2). Cry11Aa has been observed to demonstrate toxicity to *Aedes* and *Culex* larvae, with a lesser degree of activity against *Anopheles* larvae. Cry4Ba has been found to target *Aedes* and *Anopheles* larvae, but not to *Culex* larvae. Consequently, the possibility exists for the mode of toxin action to vary according to the species of mosquito as well as the type of Cry toxins. A future research direction that holds considerable interest is the investigation of the effects of sugar moieties on the mosquito-larvicidal activity using *Aedes* and *Anopheles* larvae.

GalNAc treatment may also facilitate the passage of Cry4Aa through the peritrophic membrane (PM) of *C. pipiens* larvae. The PM is a semi-permeable membrane composed of chitin and proteins and lines the entire midgut of insect larvae. In general, the PM protects the midgut from physical damage and attack from microorganisms, and it may also function as a major barrier to Cry toxins that impedes binding to receptor proteins. A previous study demonstrated that the passage of Cry1Ac through the PM is inhibited in Cry1Ac-resistant *Bombyx mori* larvae, but the passage of the toxin is restored by pretreatment with GalNAc (60).

### Conclusion

The present study demonstrates that the mosquito-larvicidal Cry4Aa exhibits a selective binding affinity for GalNAc, thereby amplifying its larvicidal efficacy against *C. pipiens*. Our data suggest that GalNAc facilitates a step associated with membrane insertion and pore formation rather than altering pore conductance. A computational analysis identified candidate GalNAc-binding cavities in the Cry4Aa architecture. However, experimental validation is necessary to confirm the role of these cavities in future studies.

## Data Availability

Data are available from the authors upon reasonable request.
